# The impact of anxiety and depression across childhood and adolescence on adverse outcomes in young adulthood: a UK birth cohort study

**DOI:** 10.1192/j.eurpsy.2023.1521

**Published:** 2023-07-19

**Authors:** I. Morales-Muñoz, P. Mallikarjun, J. Chandan, R. Thayakaran, R. Upthegrove, S. Marwaha

**Affiliations:** 1 Psychology; 2 Institute of Applied Health Research, University of Birmingham, Birmingham, United Kingdom

## Abstract

**Introduction:**

Little is still known about the long-term impact of childhood and adolescent persistent depression and anxiety on adulthood.

**Objectives:**

To investigate the impact of persistent anxiety, depression, and comorbid anxiety and depression across childhood and adolescence on the development of multiple adverse outcomes in young adulthood.

**Methods:**

This study used data from 8,122 participants in the ALSPAC cohort, in the UK. The Development and Wellbeing Assessment (DAWBA) was administered to capture child anxiety and depression symptomatology. We focused on the DAWBA generalized anxiety and mood subscales at 8, 10 and 13 years, and created a measure of comorbid anxiety and depression symptoms (i.e., anxiety + depression) at each time point. Further, a range of mental and physical health, and functional problems were assessed at 24 years. Latent Class Growth Analyses was used to detect trajectories of anxiety, depression and comorbid anxiety and depression, separately; and logistic regression to examine how persistent anxiety, depression or both associated with adverse outcomes at 24 years.

**Results:**

All three classes with persistent anxiety, depression or both associated with presenting any mental health and functional problem at 24 years. However, persistent high anxiety was not associated with any physical health problem at 24 years. Finally, high levels of comorbid anxiety and depression was the domain that exerted the greatest negative impact at 24 years.

**Conclusions:**

Children and adolescents with comorbid anxiety and depressions are at highest risk for suffering from more adverse outcomes at 24 years, compared to those presenting anxiety and depression alone.

**Disclosure of Interest:**

None Declared

